# Climate-adaptive strategies for enhancing agricultural resilience in southeastern coastal Bangladesh: Insights from farmers and stakeholders

**DOI:** 10.1371/journal.pone.0305609

**Published:** 2024-06-21

**Authors:** Md. Abdullah Al Mamun, Jianfeng Li, Aihong Cui, Raihana Chowdhury, Md. Lokman Hossain

**Affiliations:** 1 Department of Geography, Hong Kong Baptist University, Hong Kong, China; 2 Department of Food Technology and Nutrition Science, Noakhali Science and Technology University, Noakhali, Bangladesh; 3 Department of Geography and Resource Management, The Chinese University of Hong Kong, Hong Kong, China; 4 Department of Environment Protection Technology, German University Bangladesh, Gazipur, Bangladesh; University 20 Aout 1955 skikda, Algeria, ALGERIA

## Abstract

Climate change impacts crop production worldwide, and coastal regions are particularly vulnerable to its adverse effects. Given the projected rise in temperature and shifting precipitation patterns, it is crucial to examine the current challenges faced by farmers in coastal Bangladesh. Using Focus Group Discussions (FGDs) and Key Informant Interviews (KIIs), we assessed the perceptions and experiences of farmers and stakeholders regarding the existing agricultural practices, the challenges they face in crop cultivation, and the adoption of climate-adaptive practices in 2 sub-districts in the southeastern coastal region of Bangladesh. Moreover, using the Standardized Precipitation Evapotranspiration Index (SPEI) and the Standardized Terrestrial Water Storage Index (STI), we assessed the frequency and intensity of different climatic conditions in these two sub-districts. Results show that 100% of the respondents reported an increase in dry climatic conditions, the occurrence of untimely precipitation, and a decline in irrigation water during the cropping season. All the respondents in the FGDs expressed a loss of crop production because of these climate-induced disturbances. Despite these challenges, farmers have been implementing several climate-adaptive practices. Among the 9 mentioned climate-adaptive practices, 50% of FGD respondents utilize organic fertilizers, 42% cultivate heat- and drought-resilient crop varieties, use improved irrigation and harvest rainwater, and 25% cultivate integrated crops. The results of quantitative analysis of 3- and 6-month SPEI and STI values show that this region experienced frequent and intense dry climatic conditions during the growing-season, which supports the farmers’ and stakeholders’ concern about the increasing occurrence of droughts during crop growing periods. The results suggest that despite adopting climate-resilient practices under increasing growing-season droughts, farmers require support from the government and NGOs in capacity-building training and input support (e.g., stress-resilient seeds). This study holds practical implications for government, NGOs, and policymakers for ensuring sustainable agricultural productivity in the coastal region of Bangladesh.

## Introduction

Agricultural crop production is vital for global food security and plays a crucial role in meeting the food needs of the growing global population. Recent research provides quantifiable evidence of how climate change has hindered the growth of agricultural productivity on a global scale [1–3]. For example, climate variability (rise in temperature and shift in precipitation patterns) has accounted for roughly a third (32–39%) of the variations in global crop yields [4]. This highlights the adverse effects of rising temperatures, shifting precipitation patterns, and increased frequency and intensity of extreme weather events (e.g., droughts) on agricultural productivity [5]. Climate change has been observed to impact the timing and length of growing seasons [6], and these changes affect nearly 79% of wheat, 70% of maize, and 53% of rice harvesting regions of the world [4] and, thus posing significant challenges to global food security [7].

As the global population continues to grow, there is an increasing need to enhance agricultural crop production to meet the rising demand for food. Given the empirical evidence of the impacts of climate-induced stresses on crop productivity [8, 9], it is imperative to explore climate-resilient crops to maintain sustainable crop production. For example, a study conducted in India observed a 5.2% reduction in wheat yields from 1981 to 2009 due to increasing temperature [10]. Droughts are projected to have a significant negative impact on future crop yields. Tesfaye et al. [11] projected that rain-fed maize yields could decrease by an average of 3.3–6.4% by 2023 to 5.2–12.2% by 2050 because of accelerating drought intensity. Coastal regions are anticipated to face even greater challenges in crop cultivation due to the increasing occurrence of droughts, cyclones, storm surges, sea water intrusion, and sea level rise associated with climate change. The escalating frequency and intensity of droughts can lead to water scarcity, significantly affecting the availability of irrigation water for crops and resulting in reduced yields. Crop production in coastal Bangladesh has been severely affected by increasing soil and water salinity [12]. The reduction of crop yields in the coastal region of Bangladesh is expected to be even higher due to the combined effects of droughts and salinity intrusion resulting from the rise in sea level [13, 14].

Bangladesh has a long-standing history of relying on agriculture for food security and economic stability. Over the years, the country has made noteworthy progress in transitioning from an import-dependent nation to a self-sufficient one in terms of food security, particularly in agricultural crop production. For instance, Bangladesh has experienced a positive trend in rice production, with an increase from 30 million metric tons (MMT) per year in the 2000s to 35.8 MMT in 2020 [15]. The coastal regions of Bangladesh are highly susceptible to the impacts of rising sea levels due to their low-lying nature, with average elevations ranging from 1 to 4 meters above mean sea level. This vulnerability to rising sea levels is expected to escalate soil salinity in these coastal areas [13, 16]. In addition, Bangladesh is experiencing an increasing frequency and intensity of droughts [17, 18]. These drought events have exacerbated the challenges faced by the agricultural sector, resulting in a substantial decline in crop production [19] and posing a threat to the livelihoods of farmers [20]. For instance, the northwest and southwestern regions of Bangladesh have witnessed drought-related impacts on approximately 1.2 million hectares of cultivated paddy fields during the dry season [21]. Adopting drought-resilient crops can help ensure a stable food supply by enabling farmers to continue agricultural production even during water scarcity. Understanding farmers’ adoption of these crops can guide initiatives to enhance food security and reduce vulnerability to drought-induced crop failures.

Drought is a natural disaster characterized by insufficient soil moisture due to an extended absence of precipitation [22]. Assessing drought is crucial for understanding the occurrence and severity of drought events [23, 24], which, in turn, helps develop effective drought management strategies [25]. The Standardized Precipitation Evapotranspiration Index (SPEI) is widely used for drought assessment [26–28]. The SPEI can effectively identify drought events and their duration, aiding in drought management and mitigation [29]. Another important drought indicator is the Standardized Terrestrial Water Storage Index (STI), which calculates the standard deviation of the measured Terrestrial Water Storage (TWS) from the climatological average using data from the Gravity Recovery and Climate Experiment (GRACE) [22, 30].

Drought studies in Bangladesh have primarily focused on the northern part of the country. Still, it is crucial to recognize the significance of the southern region and its vulnerability to drought impacts [17, 31, 32]. The southern part of Bangladesh, situated along the coast, is responsible for most of its crop production, particularly staple food grains, due to its favorable climate conditions [33, 34]. Although this coastal region has been facing numerous climate-related challenges [16, 35], farmers cultivate crops in large quantities. The southern coastal region is not only prone to severe damages caused by cyclones and their aftermath [12, 36, 37] but is also gradually experiencing the influence of drought on agriculture [38–40]. These climate-induced disturbances affect crop production and food security in the coastal region [19, 20]. A recent study on food security in rural households in coastal Bangladesh reported that 72% of marginal landholders are food insecure [41]. These food-insecure communities will face higher crop production loss because of the projected increasing frequency and intensity of climate extremes (e.g., droughts). However, understanding how farmers and agriculture-focused stakeholders perceive the impact of drought on crop productivity in the southeastern coastal area remains unclear. Therefore, conducting studies on drought is crucial for gaining insights into the agricultural dynamics in the face of climate change in the southeastern region of coastal Bangladesh.

Understanding the perceptions of farmers, agriculture-focused service providers, and stakeholders regarding crop cultivation, climate change, and climate-adaptive crop cultivation practices is of utmost importance for promoting effective and sustainable crop production in coastal regions. Different from previous studies which were based on qualitative analysis or quantitative analysis only, this study novelly combines qualitative methodologies (FGDs and KIIs) and quantitative analysis (SPEI and STI) to evaluate agricultural resilience under climate change in a specific southern coastal area of Bangladesh. The results of this study help coastal crop producers adopt sustainable solutions. This study sought to achieve two goals by capturing respondents’ perceptions and experiences and the observed climate extremes (SPEI and STI):

- to explore the crop cultivation practices and challenges of climate-adaptive strategies employed by farmers in the southeastern coastal region of Bangladesh in response to changing climatic conditions (i.e., wet, normal, and dry); and
- to assess the temporal trends and intensity of different climatic conditions in two coastal sub-districts in Bangladesh.

This multidimensional approach provides a holistic understanding of the challenges faced by farmers in the coastal region of Bangladesh due to climate change and their adoption of climate-adaptive practices. By focusing specifically on two sub-districts in the southeastern coastal region of Bangladesh, the study provides localized insights into the perceptions, experiences, and challenges of farmers and stakeholders in this context. This helps to tailor strategies and interventions that are relevant and effective for the coastal communities in Bangladesh and other similar coastal regions worldwide.

## Materials and methods

### Profile of the study area

**Fig 1:**
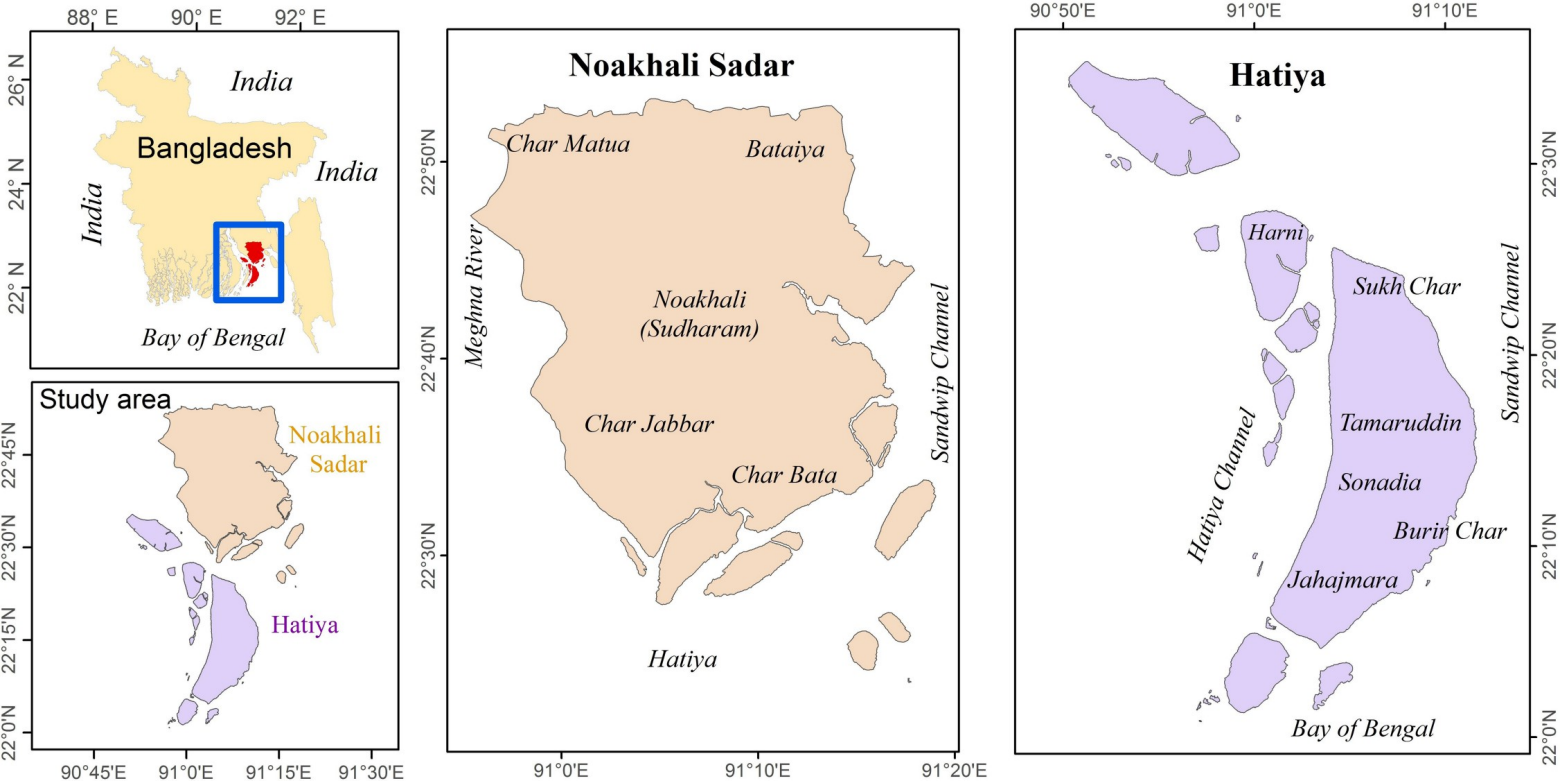
The location map of the study area (Noakhali Sadar and Hatiya sub-districts) in Noakhali district of Bangladesh.

The coastal regions of Bangladesh are highly vulnerable to natural disasters due to their geographical location and exposure to various natural hazards [38]. In this study, we selected Noakhali Sadar and Hatiya sub-districts within Noakhali district, situated in the southeastern coastal region (Fig 1). Both these sub-districts are predominantly comprised of quaternary alluvial sediments, which were deposited by the Ganges, Brahmaputra, and Meghna Rivers under tidal conditions. These deposited sediments are characterized by a dominant composition of silt and exhibit a medium to moderately fine texture [42]. These sub-districts are prone to the adverse effects of cyclones, storm surges, and sea-level rise [42, 43]. The vulnerability of these sub-districts is further exacerbated by factors such as population density, poverty, inadequate infrastructure, and limited access to early warning systems and emergency response mechanisms. With an area of 336 km^2^, Noakhali Sadar is home to a population of 0.53 million. Hatiya has a population of 0.45 million and spans an area of 1,508 km^2^. Noakhali has a high population density of 1,212 individual km^-2^, with 73,333 households, while Hatiya exhibits a lower population density of 216 individual km^-2^ and comprises 51,013 households [44]. Both sub-districts possess substantial agricultural land, with Noakhali Sadar covering an area of 92 thousand acres and Hatiya spanning 60 thousand acres. Noakhali Sadar is home to nearly 63 thousand farming households [44]. The economy of these sub-districts predominantly relies on agriculture and fishing as the primary sources of livelihood. The literacy rate is considerably low (e.g., 34% in Hatiya) compared to the national average (68%). The poverty rate in the study area (e.g., 64% in Hatiya) is higher than the national average (23%). The average annual precipitation in Noakhali Sadar is approximately 2,818 mm, while Hatiya experiences an average annual precipitation of 2,877 mm. The temperature in this region varies from 14.7°C in winter to 36°C in summer [45].

### Study design

In our study, we employed a mixed-method approach. We conducted Focus Group Discussions (FGDs) with farmers and Key Informant Interviews (KIIs) with agricultural experts to gather their insights (S1 File). This combination of qualitative methods allowed us to acquire in-depth knowledge and perspectives on agriculture-related issues. Additionally, we utilized climate data to assess the prevailing climatic conditions in the study area. By integrating qualitative and quantitative approaches, we aimed to obtain a comprehensive understanding of agricultural practices and adaptive measures under changing climatic conditions.

### Data collection

#### Qualitative data

**Fig 2:**
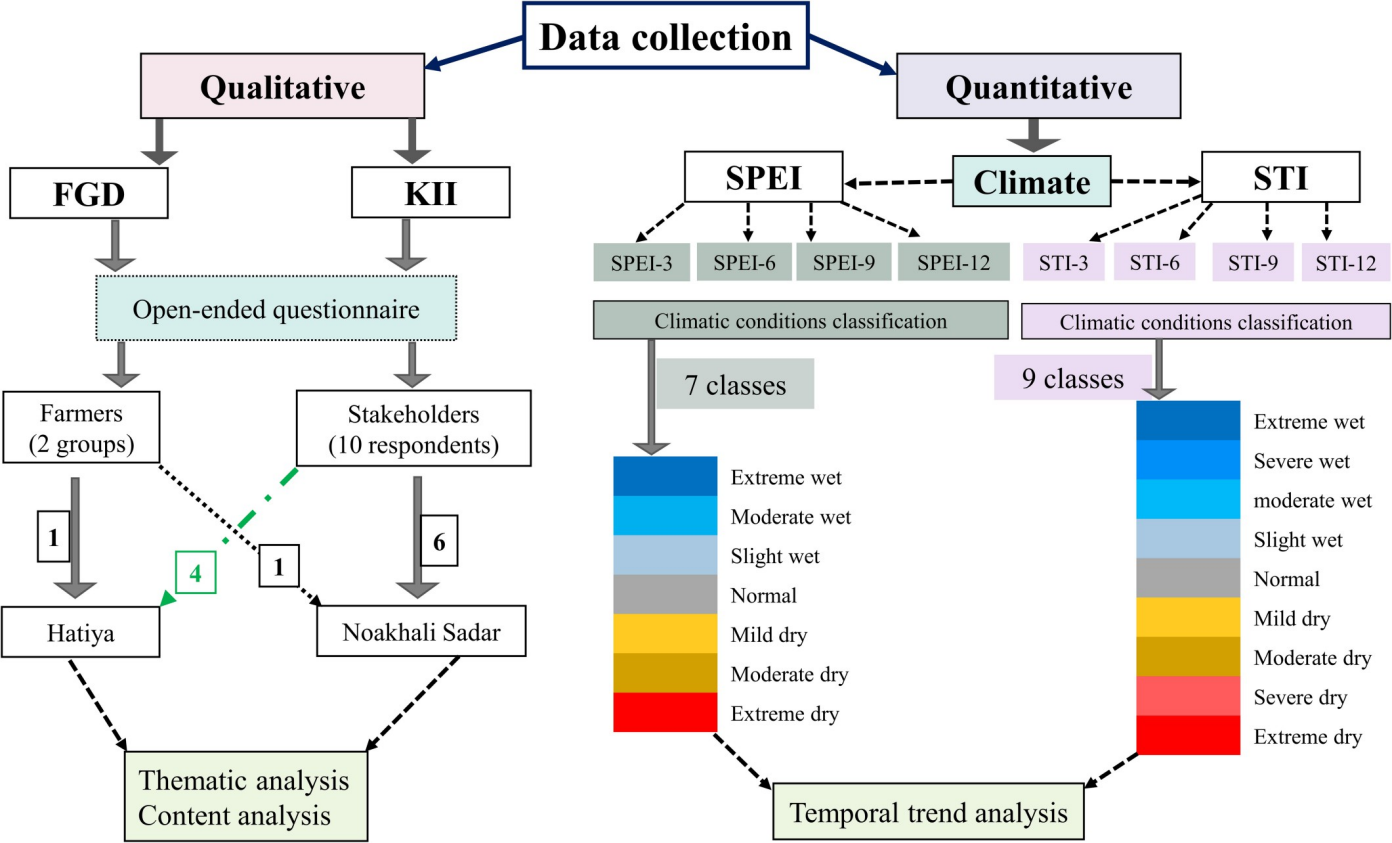
Flowchart illustrating the data collection (qualitative and quantitative), processing, and analysis. Qualitative data was obtained by conducting Focus Group Discussions (FGDs) and Key Informant Interviews (KIIs). Quantitative data includes Standardized Precipitation Evapotranspiration Index (SPEI) and Standardized Terrestrial Water Storage Index (STI) values.

We conducted 2 FGDs and 10 KIIs in Noakhali Sadar and Hatiya sub-districts for the period January-March 2020 (Fig 2). We selected the participants in FGDs and KIIs using the purposive sampling technique. We selected the participants in FGDs and KIIs. For the selection of FGD participants, we visited the Union Parishad (smallest administrative unit) and talked with the respective Union Parishad Chairman, the local elected authority leader, and Sub-Assistant Agriculture Officer. Upon receiving the farmers’ information from the Union Parishad, we selected the participants for the FGDs based on their farming experience of at least a decade. Each FGD consists of 6 male farmers (Table 1) aged between 40 and 60 years.” Specifically, we conducted 2 KIIs with professors from a public university, 6 KIIs with officers working in government line departments, and 2 KIIs with development workers affiliated with agriculture-focused NGOs (Table 1). These respondents were purposively selected based on their expertise and knowledge in agriculture. For example, both university professors are from the Department of Agriculture and have a teaching and research background on agricultural practices and their dynamics under changing climates. Additionally, respondents from the government’s agriculture-focused departments have practical experience in agricultural development and intervention projects and programs to enhance the lives and livelihoods of marginalized coastal communities. Finally, since NGOs have been supporting farmers in strengthening resilience in agriculture through capacity-building training, input support (e.g., seed and fertilizer), and farmers’ field schools (i.e., demonstration field), two KIIs were conducted with NGO representatives who have expertise in implementing agriculture-focused projects. During the KIIs, stakeholders were asked a set of questions. These included inquiries about the impacts of climate change on agriculture, climate-adaptive agricultural practices, challenges associated with adopting adaptive agriculture, and the role of policy and institutions in promoting resilient agricultural crop varieties. Each KII was 45 minutes long.

**Table 1:** The type and domain of the respondents participated in Key Informant Interviews (KIIs) and Focus Group Discussions (FGDs) during January-March 2020.

Data	Organization type	Domain	Participant’s number (profession)
KII	Education and research	University	2 (Professors)
	Government’s line-departments	Food and Agriculture	6 (Officers: Senior Scientific Officer, Scientific Officer, Upazila Agriculture Officer, Agriculture Engineer, Sub-Assistant Plant Protection Officer, and District Food Controller)
	Non-government organization	Agriculture and livelihood	2 (Officers: Agriculture and Livelihood Program Assistant and Agriculture Officer)
FGD	Farmers	Agriculture	2 groups consist of 12 farmers (active farmers: have at least a decade of farming experience)

#### Quantitative data

We obtained climate data from the Bangladesh Agriculture Research Council (BARC) [46; www.barc.gov.bd] and the Bangladesh Meteorological Department (BMD) [44; www.bmd.gov.bd]. There are two meteorological stations located in the study area. Therefore, we took into consideration both weather stations. Climate data includes monthly precipitation and the maximum and minimum temperatures of the two weather stations. In Noakhali Sadar sub-district, the weather station is known as Maizi Court station. In Hatiya sub-district, the weather station is named Hatiya station. These stations are part of the weather monitoring network in Bangladesh and contribute to the collection of meteorological data in their respective areas [45]. The climate data available at the Hatiya station spans from 1966 to 2020, while at the Maizdi Court station, it covers the period 1952–2019. We acquired climate data for the period 1991–2020 at Hatiya and 1991–2019 at Maizdi Court from the BMD. Data before 1991 was obtained from the BARC. Using the latitude and longitude of these two weather stations, we further obtained the Standardized Terrestrial Water Storage Index (STI) values for the period 2002–2017 from the Gravity Recovery and Climate Experiment (GRACE) mission [22]. As both stations fall within a single satellite image, we considered the STI values of one station, which are representative of the entire study area, including Maizdi Court and Hatiya sub-districts. The GRACE mission provides higher-resolution (0.25° x 0.25°) measurements of changes in Terrestrial Water Storage (TWS) across the globe. These measurements were then used to calculate the STI, which quantifies the deviation of TWS from its long-term average [22].

### Data processing and analysis

#### Qualitative data

Thematic and content analyses were used to process and analyze the qualitative data (i.e., FGD and KII). The thematic analysis was employed to process the long conversation sentences within the selected themes, as described by Mihas [47]. This analysis followed an inductive approach, as outlined by Joffe and Yardley [48]. The qualitative responses were initially classified to grasp their fundamental nature. Then, similar codes were organized into successive categories (axial coding), which were further developed into themes [49, 50]. During this process, the textual data was examined with a focus on the central theme titled ‘impact of climate change on crop production’. This central theme was then subdivided into three specific themes: (i) Theme-1: climate change and traditional agriculture, (ii) Theme-2: climate adaptive agriculture, and (iii) Theme-3: food security. We searched within the transcribed textual content for these three subdivided themes. Theme-1 aimed to gather valuable insights from the respondents of FGDs and KIIs regarding the effects of climate change on agricultural systems. By exploring this theme, we sought to gain a deeper understanding of how climate change is impacting various aspects of agricultural practices, production, and overall agricultural systems. Within Theme-2, we aimed to explore the various strategies and techniques farmers have been implementing or considering to adapt their agricultural practices to the changing climatic conditions. In this Theme, we also sought to gather insights from the respondents in KIIs regarding their understanding of agricultural adaptation, the interventions of government and NGOs in promoting climate-resilient agriculture, and the research conducted to find improved seed varieties for addressing climate change impacts. The focus of Theme-3 was to understand respondents’ perceptions regarding the potential risks of crop production loss and the subsequent impacts on the dietary and nutritional quality of food.

Furthermore, we employed content analysis to examine the textual data gathered from the FGDs with farmers [51]. To do so, we extracted information related to agricultural adaptation techniques mentioned by the respondents in the transcripts of the FGDs. During the analysis of the FGD transcripts, several frequently mentioned adaptive techniques were identified. These techniques included harvesting rainwater, using pheromone traps, organic fertilizer, integrated cropping, sorjan methods, improved seed varieties, irrigation, and mulching. Despite the respondents’ awareness of these techniques, it was observed that not all farmers were utilizing them in their farming operations. By quantifying the number of farmers who were aware of these adaptive techniques and the number of farmers who reported using them, we aimed to assess the adoption and implementation of these strategies in the study area. This analysis helped identify the gap between awareness and implementation, shedding light on potential barriers or challenges farmers face in adopting these techniques.

#### Quantitative data

We calculated Standardized Precipitation Evapotranspiration Index (SPEI) values for the period 1952–2019 for Maizdi court station and 1966–2020 for Hatiya station using the obtained temperature and precipitation data from the BARC and BMD. The calculation of SPEI values was performed using the Hargreaves method in the statistical software R [52]. To do so, we used the monthly maximum and minimum temperature, monthly precipitation data, and the latitude of the study stations to calculate the monthly Potential Evapotranspiration (PET) values using the Hargreaves method. Then, the monthly water balance was derived by subtracting PET from precipitation values. Finally, SPEI values for different time scales were computed using the monthly water balance. We utilized four different time scales of SPEI values: 3-month (SPEI-3), 6-month (SPEI-6), 9-month (SPEI-9), and 12-month (SPEI-12). To assess the climatic conditions, we focused on the SPEI values for August, which represent the end of the growing season. For example, the SPEI-3 values for August denote the climatic conditions for June-August. Likewise, the SPEI-6, SPEI-9, and SPEI-12 values for August indicate the climatic conditions for March-August, December-August, and September-August, respectively. The SPEI values were then classified into 7 classes ranging from extreme wet (SPEI values ≥ 1.50) to normal (SPEI values −0.49 to ≤ 0.49) to extreme dry (SPEI values ≤ −1.50) conditions (see Table 2A for classification), based on an established climatic condition classification (Fig 2) [53].

**Table 2:** Climatic condition classification based on (a) the Standardized Precipitation Evapotranspiration Index (SPEI) proposed by Nam et al. [53] and (b) the Standardized Terrestrial Water Storage Index (STI) developed by Cui et al. [22].

(a) SPEI		(b) STI	
Climatic condition	SPEI value	Climatic condition	STI value
Extreme/severe wet	≥1.50	Extreme wet	≥ 2.0
Moderate wet	1.00 to ≤ 1.49	Severe wet	1.5 ~ 2.0
Slight wet	0.50 to ≤ 0.99	Moderate wet	1.0 ~ 1.5
Normal	-0.49 to ≤ 0.49	Slight wet	0.5 ~ 1.0
Mild dry	-0.99 to ≤ - 0.50	Near Normal	−0.5 ~ 0.5
Moderate dry	-1.49 to ≤ -1.00	Slight Dry	−1.0 ~ -0.5
Extreme/severe dry	≤ - 1.50	Moderate dry	−1.5 ~ -1.0
		Severe dry	−2.0 ~ -1.5
		Extreme dry	≤−2

To check the robustness of climatic conditions, we further used monthly STI values of the study region for the period 2002–2017. The monthly STI values obtained from the GRACE mission represent standardized measurements of terrestrial water storage anomalies from 2002–2017. These anomalies reflect deviations from the long-term average conditions, allowing for identifying both positive and negative variations in water storage [22]. Similar to SPEI time scales, we used four distinct time scales of STI values to assess climatic conditions for August. These STI time scales included 3-month (STI-3), 6-month (STI-6), 9-month (STI-9), and 12-month (STI-12). The STI-3 values of August represent the climatic conditions of June-August. The obtained STI values were categorized into 9 classes representing a range of climatic conditions. These classes spanned from extreme wet (STI values ≥ 2.0) to near normal (STI values −0.5 ~ 0.5) to extreme dry (STI values ≤ −2) conditions (see Table 2B for classification), based on a newly developed climatic condition classification [Fig 2; 22]. The STI values act as indicators of hydrological drought, while SPEI values serve as indicators of meteorological drought.

In quantitative analysis, first, we assessed the temporal trends of SPEI and STI values for 4 distinct time scales (i.e., SPEI-3 to SPEI-12, and STI-3 to STI-12) for the study region. The Kendall trend test was applied to assess the trends of SPEI values for 1952–2019 at Maizdi court and 1966–2020 at Hatiya stations and STI values over 2002–2017 for entire study regoin [54]. Second, we counted the number of specific climatic conditions (e.g., extreme dry, normal, and extreme wet) from the SPEI and STI values for each time scale (e.g., SPEI-3) and plotted the number of events using the ‘ggplot2’ package in R to display the frequency of different climatic conditions [52]. Finally, using the Kendall trend test, we further assessed the temporal trends of annual minimum SPEI and STI values for the four-time scales to evaluate the increasing or decreasing trends of climatic conditions in respective weather stations for SPEI and in the entire study region for STI.

### Ethical considerations

The research ethics board at Noakhali Science and Technology University approved our study, with the ethical approval number NSTU/Reg/Estab/Research cell/2020/7839. We adhered to the code of ethics outlined in the World Medical Association Declaration of Helsinki concerning the involvement of human subjects. Participants selected for the focus group discussions and key informant interviews were provided with clear information about the study’s objectives. The written consent was taken from the FGD and KII respondents. They were assured that their opinions, information, and concerns would remain confidential and anonymous and that their names and identities would not be used in reporting and analysis.

## Results and discussions

### Perceptions of the respondents

The textual data of the perceptions of the respondents in FGDs and KIIs were assigned to a central theme (impact of climate change on crop production), which was sub-divided into 3 specific themes. Theme-1 (climate change and traditional agriculture) aimed to gather insights from the respondents in FGDs and KIIs on the effects of climate change on agricultural systems, providing a deeper understanding of its impacts on various aspects of agricultural practices and production. Theme-2 (climate adaptive agriculture) focused on exploring respondents’ strategies for adapting their practices to changing climatic conditions, including their understanding of agricultural adaptation, interventions by government and NGOs, and research on improved seed varieties. Theme-3 (food security) centered on understanding respondents’ perceptions of the risks of crop production loss and the subsequent effects on the dietary and nutritional quality of food. The key points of the respondents’ perceptions under each theme have been explained below:

### Theme-1 (climate change and traditional agriculture)

Farmers traditionally employed specific methods for cultivating agricultural crops. One common practice among farmers is monoculture, which focuses on cultivating a single crop type. For instance, paddy cultivation has been a prevalent practice in both sub-districts. However, KII respondents have been experiencing gradual changes in climatic conditions and a decline in soil fertility. The availability of irrigation water has become a pressing issue, significantly impacting paddy cultivation. Shortages in irrigation water have posed challenges for farmers, leading to difficulties in maintaining adequate water supply for the paddy fields. The impact of irrigation water shortage on paddy cultivation was reported in both FGDs. While discussing the precipitation and crop cultivation, a respondent in a FGD in Hatiya sub-district (FGD-2) stated that:

*“We struggle with precipitation shortage during our crop growing season*. *We also face frequent and intense natural disasters*, *especially cyclones in April-May and October-November*. *The untimely precipitation*, *frequent cyclones*, *storm surges*, *and monsoon floods affect our agricultural productivity*. *We are trying to cope with this common natural disaster*. *However*, *in recent years*, *we are facing a great challenge in crop cultivation because of repeated droughts and heatwaves”*.

While discussing with a KII respondent (agriculture sector) in Hatiya sub-district (KII-3), the responses of the FGD respondent were also reflected, who expressed that:

*“Increasing temperature is the main reason for climate change*. *Firstly*, *most of the agricultural sector follows the monoculture technique*, *where lands are mostly used for paddy cultivation*. *Secondly*, *reservoirs are not being preserved to hold a lot of water*. *As a result*, *ground water layers are slowly falling*, *hampering our agricultural work*. *Seawater enters the freshwater reservoir*, *which becomes useless for agriculture*. *The fertility of land also decreases because of these reasons”*.

Given the challenges highlighted by the FGD and KII respondents, cultivating drought-tolerant crops becomes imperative to mitigate the adverse effects of decreasing groundwater levels and salinization, ensuring sustainable agricultural practices in the studied areas. Recognizing these challenges, the Bangladesh government has launched several interventions to tackle drought-induced crop losses and promote resilience in the agricultural sector. The government’s interventions have been reported during the conversation with a KII respondent (university teacher: KII-1), who stated that:

*“There are 33 Agro Ecological Zone in Bangladesh*. *Every district is fond of growing different crops because of different climates*. *Nearly one-quarter of our land is not cultivated due to a lack of soil moisture*. *Farmers are trying to grow new varieties of crops in water-stressed soil*, *but the yields are being affected because of recurrent occurrences of drought in recent years*. *The Department of Agriculture Extension of Bangladesh’s government*, *universities*, *and research organizations have introduced many interventions for improving crop productivity*. *For example*, *in recent years*, *farmers have been cultivating drought-tolerant crops*, *such as watermelon*, *cucumber*, *frog fruit*, *country bean*, *and sunflower”*.

The evidence provided by the FGD and KII respondents highlights the impact of climatic variability on agricultural crop cultivation in the region. The traditional cultivation of paddy has been significantly affected due to untimely precipitation and water shortages during the growing season, resulting in decreased crop production. Similar evidence was reported in several studies [31, 36] conducted in coastal regions of Bangladesh. For example, assessing the agricultural livelihood vulnerability in coastal regions under climate change, Hoque et al. [31] showed that southeastern coastal regions, including our study region (Hatiya and Noakhali Sadar sub-districts), are most vulnerable to climate change. Droughts in the paddy growing season in Bangladesh were reported by Prodhan et al. [55], who showed that severe to extreme drought was more frequent during the paddy (Boro rice) growing season. These increasingly intense droughts affect crop production in many coastal districts and make the community’s livelihood vulnerable [56]. In response to these challenges, the government is actively enhancing agricultural production by promoting resilient crop seeds [57]. Climate-adaptive agriculture is seen as a viable option in Bangladesh in the face of the increasing magnitude and strength of climate extremes, which has been explained in our Theme-2.

### Theme-2 (climate-adaptive agriculture)

Climate-adaptive agriculture plays a pivotal role in maintaining production and promoting sustainable agriculture, particularly in the face of increasing climate-induced shocks [58]. The FGD and KII respondents in our study reported several climate-adaptive agricultural measures that farmers have been practicing in recent years. These measures include cultivating drought- and heat-tolerant crops, implementing efficient irrigation techniques, diversifying crop varieties, improving soil health, and integrating agroforestry and conservation practices. A respondent in an FGD in Hatiya sub-district (FGD-2) who is also cultivating climate-adaptive crops mentioned that:

*“We have been facing several natural disasters*, *among which cyclones*, *floods*, *and soil erosion were prominent*. *But in recent decades*, *we have been facing a new type of disturbances*, *particularly extreme drought*, *heatwaves during summer*, *intrusion of saline water into freshwater bodies*, *and outbreaks of pests*. *Because of these challenges*, *we are getting less production*. *However*, *with the support of the Department of Agriculture Extension in our government*, *we received capacity-building training and participated in climate field school (crop demonstration field)*. *Some of our farming communities receive heat- and saline-tolerant seeds from the agriculture department*. *Climate-resilient crops*, *particularly potato*, *mustard*, *sunflower*, *and rice (BINA 10 variety)*, *are growing in popularity among marginalized farmers*. *To meet the irrigation water*, *we use harvested rainwater and to control pests*, *we use a Pheromone trap”*.

By adopting climate-resilient practices and technologies, farmers can effectively mitigate the adverse impacts of climate change on agricultural systems. Emphasizing climate-adaptive agriculture helps safeguard crop yields and food security and enhances the resilience of farming communities in the face of ongoing climate challenges. While several climate-adaptive agriculture techniques have been reported by the farmers (e.g., FGD-2) in our study areas, further research is needed to explore the resilient crop varieties. The dynamic nature of climate change requires continuous efforts to develop crops that can withstand and thrive in changing environmental conditions, which was also emphasized by a respondent of a KII (university teacher: KII-2), who stated that:

*“Growing season climatic conditions have been shifting due to climate change*. *Farmers receive less rainfall during the crop growing season while receiving high rain during pre-harvest time*. *This imbalance of seasonal rainfall*, *along with temperature fluctuation*, *affects their crop production*. *Considering the country-wide evidence of climate-induced shocks on crop production*, *agriculture department*, *and universities have been trying to invent new varieties of climate-friendly crops such as saline-*, *heat-*, *and pest-tolerant crops*. *Bangladesh government has already invented several high-yielding and climate-resilient crops (e*.*g*., *BINA 7 and 10 paddy*, *sunflower*, *and soybean)”*.

The insights shared by respondents in both the FGD and KII strongly emphasize the profound impact of climatic variability on crop cultivation in our study region (Theme-2). These findings explain the vulnerability of agricultural systems to the changing climate and highlight the urgent need for adaptation measures. The adoption of sustainable practices in our study region, such as rainwater harvesting for irrigation and the use of Pheromone traps for pest control, demonstrates the resilience and adaptability of farming communities in responding to the changing climate. These practices address water scarcity challenges, contribute to environmental conservation, and promote sustainable agriculture. The distribution of heat- and saline-tolerant seeds by the agriculture department indicates a proactive approach to promoting crop varieties that can withstand the adverse effects of climatic variability. Like our findings in Theme-2, several empirical studies supported that climate-adaptive agricultural practices enhance diverse possibilities of increasing crop yield, strengthening livelihood, and enhancing social resilience [59–61].

The efforts of the Bangladesh government in inventing high-yielding and climate-resilient crops, such as BINA 7, BRRI 71, and BRRI 56 paddy, maize, sunflower, and soybean, are noteworthy [58]. These varieties have been specifically bred or selected to withstand the challenges posed by climate change, including extreme temperatures, saline intrusion, and pest outbreaks. The findings in Theme-2 highlight the importance of climate-adaptive agriculture as a response to the adverse impacts of climate change on crop production. By developing and promoting climate-friendly crop varieties, farmers can enhance their resilience and adaptability to changing climatic conditions [62]. These climate-adaptive crops offer the potential to mitigate the adverse effects of climatic variability, such as water scarcity, increased salinity, and pest infestations [14]. The active involvement of the agriculture department and universities in inventing and disseminating climate-resilient crop varieties showcases their proactive stance in addressing climate change in the agricultural sector [63]. This focus on climate-adaptive agriculture not only ensures food security and maintains agricultural productivity but also supports the resilience and livelihoods of farmers, paving the way for a more sustainable and resilient future.

### Theme-3 (food security)

Understanding food security at the community level is crucial, particularly in changing climate conditions (Theme-3 in our qualitative study). Food security refers to the availability, accessibility, and utilization of sufficient, safe, and nutritious food to meet the dietary needs and preferences of individuals within a community. With changing climate patterns, the stability and predictability of agricultural production are increasingly challenged. Extreme weather events, shifts in growing seasons, and altered precipitation patterns can significantly impact crop yields and agricultural productivity. These changes can disrupt food availability and pose risks to the food security of communities, particularly those heavily reliant on local agricultural production. In our study region, a large portion of the population relies heavily on crops for their livelihoods, emphasizing the crucial role of food security. The high poverty levels in the Hatiya and Noakhali sub-districts indicate that many individuals may lack the financial resources to ensure consistent access to an adequate and diverse diet. Limited income can restrict their ability to purchase a variety of nutritious foods, leading to a heavy reliance on staple crops or carbohydrates for sustenance. This overemphasis on a single food group can result in nutritional deficiencies and negatively impact overall health and well-being, which is reflected in a conversation with a KII respondent (university teacher: KII 2), who stated that:

*“Food utilization plays a crucial role in promoting human health*, *yet many individuals*, *particularly in Asian countries like Pakistan*, *India*, *and Bangladesh*, *are unaware of the importance of a diverse and balanced diet*. *Rice*, *a staple in these regions*, *primarily consists of starch and lacks essential vitamins and minerals*. *To maintain a healthy lifestyle*, *individuals of all ages*, *from infants to the elderly*, *should consume an average of 220 grams of vegetables daily to meet their nutritional needs*. *Unfortunately*, *due to a lack of proper dietary knowledge*, *people tend to rely more on rice than vegetables*. *It is crucial for individuals to understand the nutritional value of different foods and make informed choices*. *However*, *access to fruits and vegetables remains limited*, *especially in coastal regions like Hatiya and Noakhali Sadar sub-districts*, *where climate-induced shocks and poor soil conditions make it challenging to cultivate such crops”*.

Natural calamities threaten the availability and access to an adequate and diverse food supply. The decreased agricultural production resulting from excessive or insufficient rainfall disrupts the local food production systems, making it difficult for communities to meet their dietary needs. The scarcity of nutritious foods further exacerbates the problem, as these food items are essential sources of vitamins, minerals, and protein. The food insecurity issue in coastal Bangladesh has been documented in a recent study [41], which reported that 72% of marginal landholders are food insecure. Our study respondents emphasize the importance of implementing strategies to enhance the resilience of agricultural systems and ensure a stable food supply. While explaining the dietary behavior and food nutrition, a KII respondent (KII-7) stated that:

*“Excessive rainfall during June-August makes crop harvesting challenging*, *leading to decreased agricultural production*. *Conversely*, *insufficient irrigation facilities during the flowering stage of winter crops further reduce crop yields*. *These climate-related factors contribute to a limited supply of nutritious foods*, *including fruits and vegetables*. *Consequently*, *the nutrition status in the area is critically low*, *and the population faces significant malnutrition challenges”*.

The low nutrition status in the area highlights the urgent need to address food security challenges comprehensively. Efforts should focus on mitigating the impact of natural calamities through climate-resilient agricultural practices. This can include techniques such as improved water management, crop diversification, and resilient crop varieties. Crop diversification is a key strategy to enhance food security and nutrition status in the community. By cultivating a wide range of crops, farmers can reduce their dependence on a single crop and minimize the risks associated with crop failure due to adverse weather conditions. However, poverty remains a significant barrier to accessing nutritious food. Many individuals and families lack the financial resources to afford a diverse and balanced diet. As a result, they are often unable to meet their nutritional needs, leading to malnutrition and related health issues, which have been documented from a respondent in an FGD in Noakhali Sadar sub-district (FGD-1), who mentioned that:

*“Due to financial constraints*, *our diet primarily relies on locally grown food options*. *As a result of our lack of education*, *we are not fully aware of the nutritional value of different foods*. *While we understand the importance of protein in our diet*, *our poverty prevents us from affording protein-rich sources such as meat*, *legumes*, *vegetables*, *and fish”*.

The insights shared by respondents in the FGDs and KIIs highlight the significant influence of climatic variability on food security and nutritional status in our study region. The changing climate patterns profoundly impact agricultural productivity, crop yields, and the availability of nutritious food sources. The respondents expressed concerns about the adverse effects of extreme weather events, which often lead to crop failures and food shortages. These challenges directly contribute to food insecurity. Like the statements of the respondents in our FGDs and KIIs, similar findings were also reported in several other studies in other coastal districts [41, 64, 65]. For example, Islam et al. [65] reported that climate-induced shocks have significant negative impacts on crop failure, food security, and household basic food consumption. Similarly, Panezai et al. [41] reported that landless farm households are at high risk of food insecurity because of increasing heavy precipitation and flood events as well as soil and water salinity.

The lack of awareness regarding the importance of a diverse and balanced diet is a significant issue, particularly in areas where rice is a staple food. Rice, while a filling carbohydrate source, lacks essential vitamins and minerals for optimal health. However, the limited knowledge about dietary practices often leads to an overreliance on rice and a neglect of vegetables. This knowledge gap needs to be bridged through comprehensive nutrition education programs emphasizing the importance of a varied and nutrient-rich diet. Assessing the linkage between agriculture and nutrition and dietary diversity, Headey and Hoddinott [66] reported that despite the rapid growth in rice productivity in Bangladesh, there has been relatively sluggish diversification in both food production and consumption. One main reason for the sluggish diversification of food consumption is financial constraints, which has been reported by our FGD and KII respondents. This evidence also supports the findings of other similar studies in coastal districts in Bangladesh [67, 68], which explored that financial constraints impact dietary diversity and food accessibility of households in the southwestern coastal region of the country. This limited dietary diversity further contributes to nutritional deficiencies and hampers efforts to improve food security and nutrition. Addressing the challenges of food security and nutrition requires a comprehensive approach that encompasses nutrition education, climate-resilient agricultural practices, improved irrigation facilities, and poverty alleviation measures. Addressing these interrelated factors makes it possible to improve the nutrition status and overall well-being of the population in the mentioned regions and beyond.

### Understanding the dynamics of climate-adaptive techniques

Using content analysis of the FGD script, we assessed the respondents’ level of awareness and utilization of climate-adaptive techniques in their farming practices (Table 3). The results show the number of respondents who were aware of each technique and those who used the technique. Among the respondents, the highest level of awareness was observed for organic fertilizer, irrigation, rainwater harvest, drought-tolerant crop cultivation, and improved seed variety, with 8, 8, 7, 7, and 7 aware respondents, respectively. These techniques have gained significant attention and recognition among the surveyed individuals. Regarding utilization, organic fertilizer, and irrigation were the most commonly used techniques (with 6 and 5 users, respectively). Rainwater harvest and drought-tolerant crop cultivation were utilized by 5 respondents each, while 4 respondents used improved seed variety. The use of pheromone traps, integrated cropping, application of the Sorjon method, and mulching had relatively lower levels of utilization, with 3, 3, 2, and 2 users, respectively.

**Table 3:** Awareness and utilization of climate-adaptive techniques obtained from content analysis of 2 Focus Group Discussions (FGDs) in Noakhali Sardar and Hatiya sub-districts in coastal Bangladesh.

Name of technique	No. of aware respondents (% of total respondents)	No. of user respondents (% of total respondents)
Rain water harvest	7 (58)	5 (42)
Use of pheromone trap	6 (50)	3 (25)
Organic fertilizer	8 (67)	6 (50)
Integrated cropping	5 (42)	3 (25)
Drought-tolerant crop cultivation	7 (58)	5 (42)
Application of Sorjon method	3 (25)	2 (17)
Improved seed variety	7 (58)	4 (33)
Irrigation	8 (67)	5 (42)
Mulching	3 (25)	2 (17)

The results highlight the varying degrees of familiarity and adoption of climate-adaptive techniques among the FGD respondents. Among the respondents, drought-tolerant crop cultivation, organic fertilizer, rainwater harvest, irrigation, and improved seed variety emerged as the most well-known techniques. This indicates that these techniques have gained significant attention and recognition among the participants, suggesting their perceived importance in adapting to climate change in agricultural practices. Regarding utilization, drought-tolerant crop cultivation, organic fertilizer, rainwater harvest, and irrigation were the most commonly employed techniques. This observation aligns with the high awareness levels of these techniques, indicating a positive correlation between awareness and utilization. The growing awareness and adoption of drought-tolerant crop cultivation have been reported in several other regions in Bangladesh. For example, Rahman et al. [20] reported that adoption of drought-tolerant rice varieties is increasing, leading to higher yields and income for farmers. This climate-adaptive technique seems viable considering the loss of crop productivity due to increasing droughts. Bangladesh’s government is promoting this technique as this country experienced an extreme drought in 1999, which reduced 25–30% of crop production [21]. More than half of the country’s cultivable land has experienced moderate to extreme drought in recent decades [21]; thus, drought-resilient crop varieties are growing in popularity to maintain stable crop production.

Like drought-resilient crops, organic fertilizer is also getting greater attention among marginalized farmers. For example, in drought-prone and salinity-affected regions, farmers are using organic fertilizer (e.g., vermicompost: produced from cowdung and organic waste) [69, 70], as many empirical evidence showed positive effects of vermicompost on improving soil nutrients and moisture retention capacity and crop productivity [71, 72]. To tackle the drought impacts, rainwater storage in the reservoir (e.g., pond and small ditch) is another adaptive technique in crop production reported by the respondents of our FGDs, which is consistent with Islam [73], who reported that farmers in the southwestern coastal region (Baerhart district) use harvested rainwater in irrigating their crop fields.

### Assessment of climatic conditions

#### Assessment of climatic conditions by SPEI

**Fig 3:**
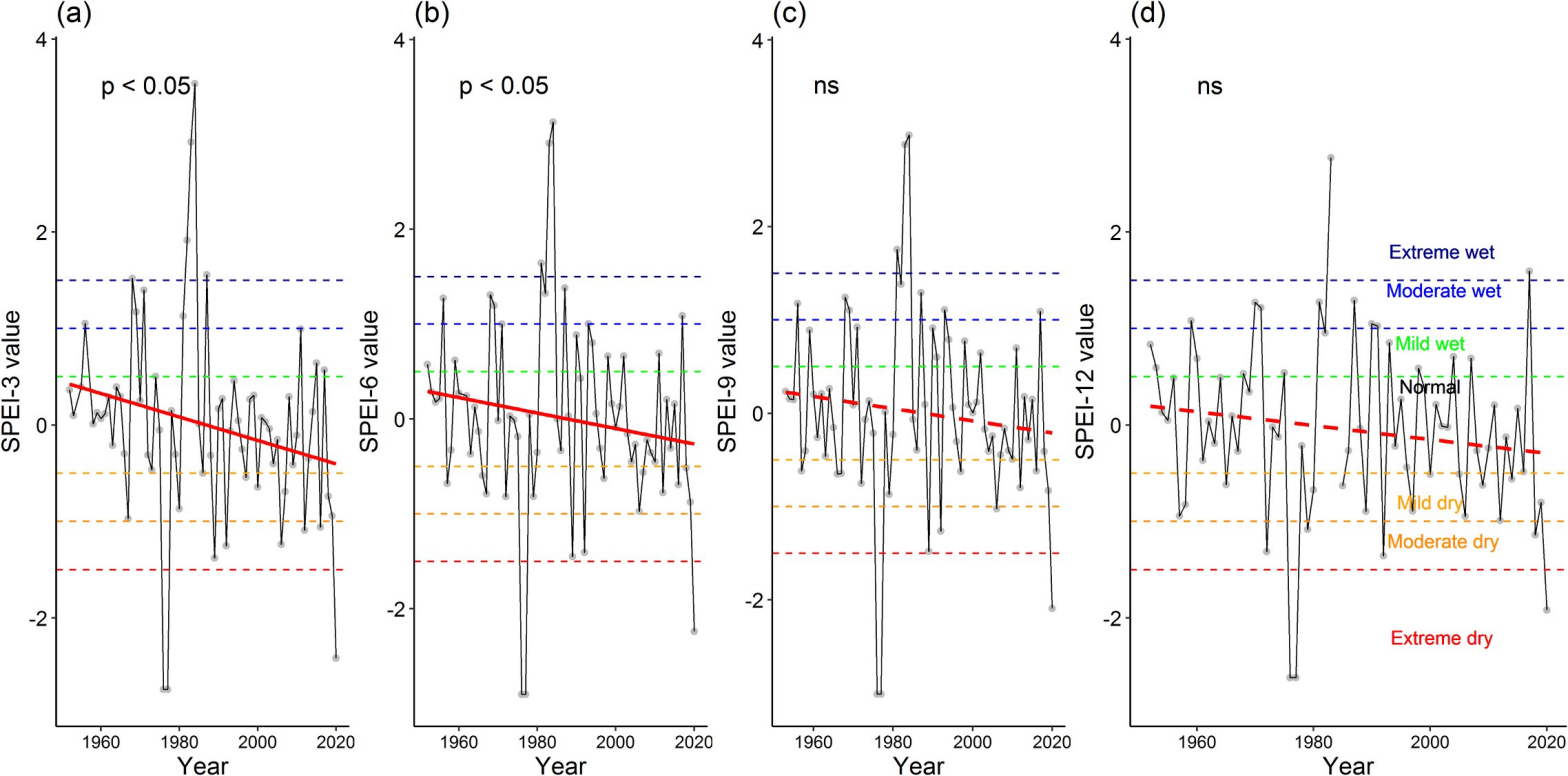
Temporal trend of Standardized Precipitation Evapotranspiration Index (SPEI) values for (a) SPEI-3, (b) SEPI-6, (c) SPEI-9, and (d) SPEI-12 at Maizdi court weather station for the period 1952–2019. The *p-value* of the significance of the trend from the Kendall trend test is shown. The symbol ‘ns’ indicates the insignificance of the trend.

**Fig 4:**
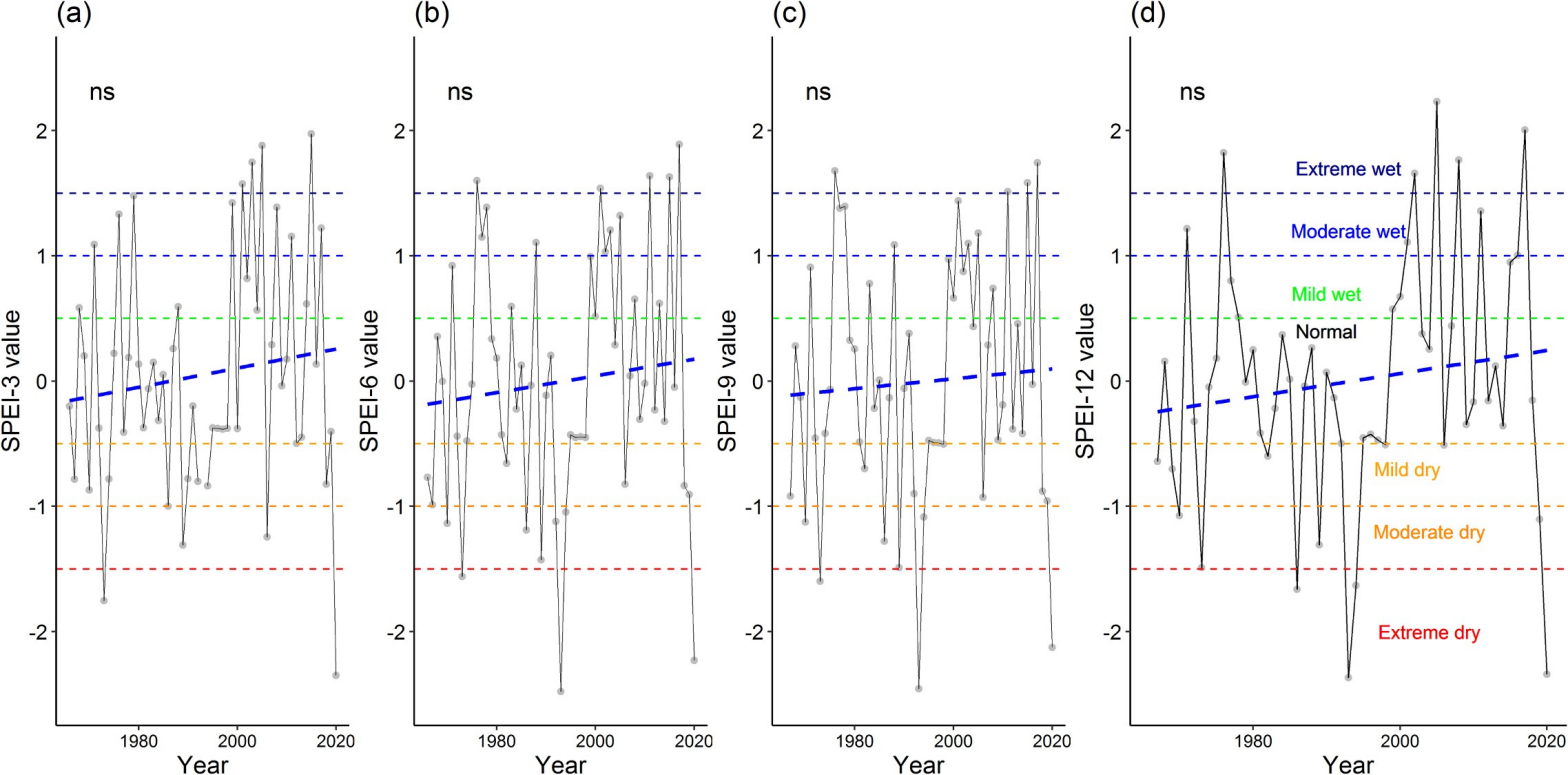
Temporal trend of Standardized Precipitation Evapotranspiration Index (SPEI) values for (a) SPEI-3, (b) SEPI-6, (c) SPEI-9, and (d) SPEI-12 at Hatiya weather station for the period 1966–2020. The symbol ‘ns’ indicates the insignificance (p > 0.05) of the temporal trend of SPEI values obtained from the Kendall trend test.

Using the SPEI values of respective weather stations, we assessed the temporal trends of SPEI values. The results show the differential patterns of the trends of climatic conditions in two weather stations (Figs 3 and 4). Specifically, the SPEI values showed a decreasing trend for the Maizdi court weather station, of which SPEI-3 and SPEI-6 values significantly decreased for the period 1952–2019 (both *p* < 0.05; Fig 3A and 3B). The decreasing trends of the SPEI-9 and SPEI-12 values in this station were insignificant (*p* > 0.05; Fig 3C and 3D). Surprisingly, SPEI values for the 4 studied time scales (i.e., SPEI-3 to SPEI-12) showed an increasing trend, albeit insignificantly, for the period 1966–2020 at Hatiya weather station (all *p* > 0.05; Fig 4).

**Fig 5:**
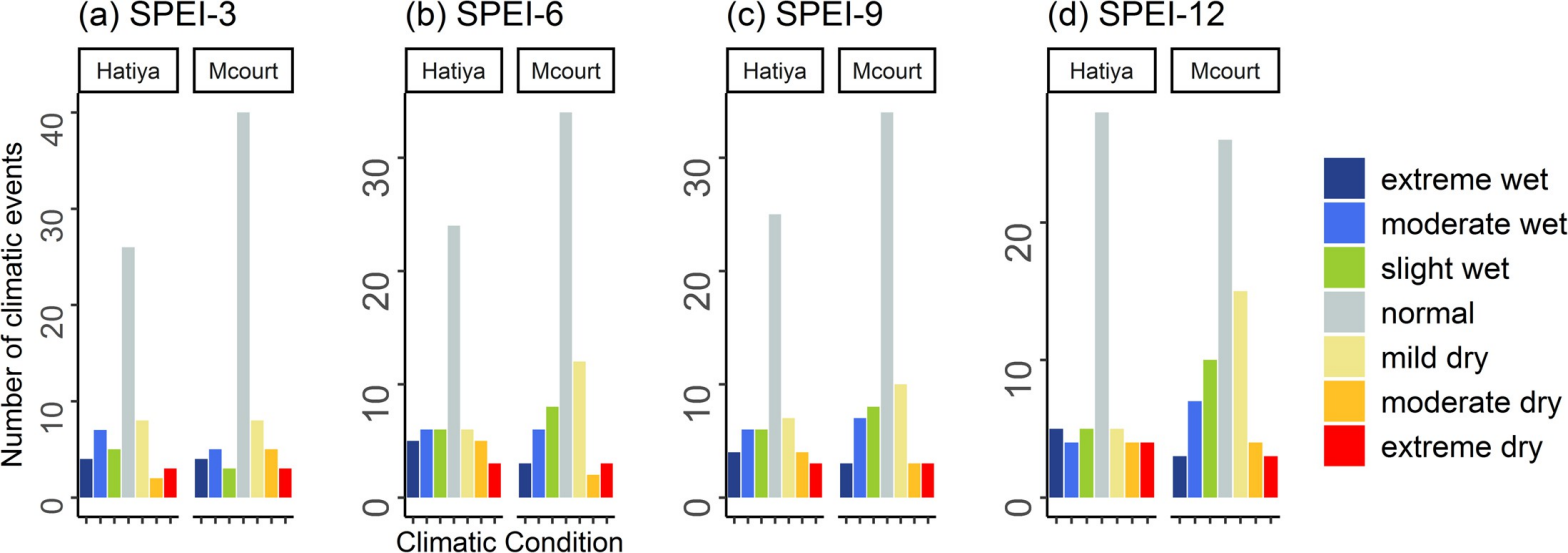
The frequency of 7 climatic conditions (extreme wet, moderate wet, slight wet, normal, mild dry, moderate dry, and extreme dry) based on Standardized Precipitation Evaporation Index (SPEI) values for four-time scales: (a) SPEI-3, (b) SPEI-6, (c) SPEI-9, and (d) SPEI-12 at Hatiya and Maizdi court weather stations.

The analysis of climatic conditions using SPEI values at both Hatiya and Maizdi court weather stations revealed a range of wet and dry events, spanning from extreme wet to extreme dry conditions. For the SPEI-3 values, the Hatiya station experienced 14 dry events and 16 wet events (Fig 5A). Considering the SPEI-6 values, this station recorded 14 dry events and 17 wet events over the past 55 years (Fig 5B). This station recorded 15 dry events and 16 wet events for SPEI-9 values (Fig 5B), and 14 dry events and 14 wet events for SPEI-12 values (Fig 5D). At the Maizdi court station, over the past 71 years, there were 15 dry events, categorized as 7 mild, 5 moderate, and 3 extreme dry, based on the SPEI-3 values (Fig 5A). Additionally, there were 13 wet events, consisting of 3 slight, 5 moderate, and 5 extreme wet conditions for the same period (Fig 5A). Similarly, when considering the SPEI-6 values, the Maizdi court station recorded 16 dry events, including 11 mild, 2 moderate, and 3 extreme dry conditions (Fig 5B). Additionally, there were 17 wet events, comprising 7 slight, 7 moderate, and 3 extreme wet conditions (Fig 5B).

These findings indicate that both stations experienced a similar pattern of dry and wet events, although the exact frequencies and intensities varied. The occurrence of dry and wet events underscores the potential challenges faced by agricultural activities in these regions, as water availability and precipitation patterns play a crucial role in crop growth and productivity. Notably, it is evident that, on average, every 2.5 years, Maizdi Court station experienced either a dry or wet event during the growing season, as indicated by the SPEI-3 and SPEI-6 values over the past 71 years. For Hatiya station, almost every other year during the growing season, the station encountered either dry or wet events as indicated by the SPEI-3 and SPEI-6 values.

**Fig 6:**
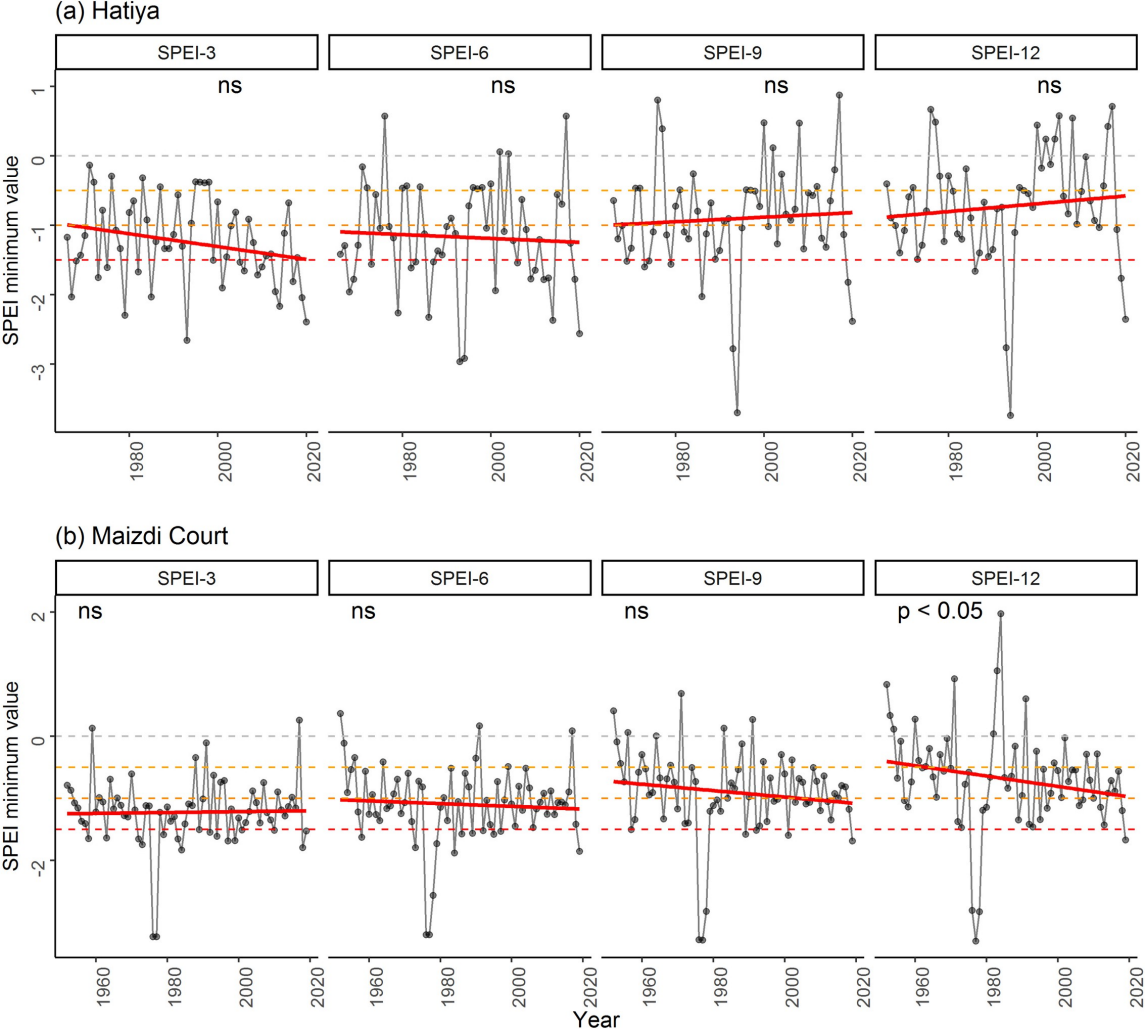
Temporal trends of Standardized Precipitation Evapotranspiration Index (SPEI) minimum values of 4 time scales (i.e., SPEI3, SEPI6, SPEI9, and SPEI12) at (a) Hatiya and (b) Maizdi court weather stations. The *p-value* of the significance of the trend from the Kendall test is shown. The symbol ‘ns’ indicates the insignificance of the trend.

We further assessed the temporal trend of the intensity of dry conditions using minimum SPEI values for both stations (Fig 6). These minimum SPEI values did not show any detectable patterns for 4 time scales (Fig 6), except for a significant decreasing trend of minimum SPEI-12 values at Maizdi Court station (p < 0.05; Fig 6B).

#### Assessment of climatic conditions by STI

**Fig 7:**
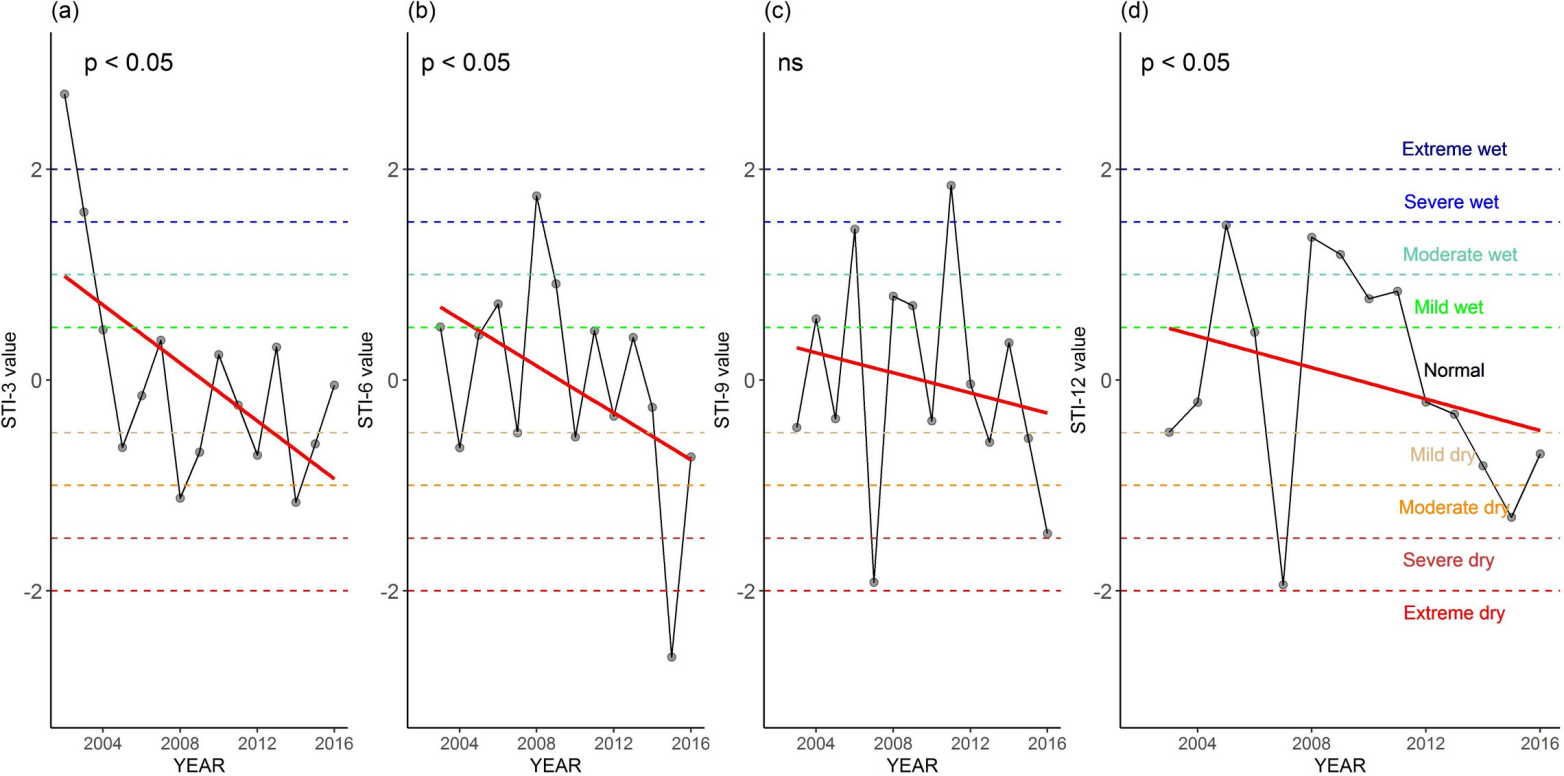
Temporal trend of Standardized Terrestrial Water Storage Index (STI) values for (a) STI-3, (b) STI-6, (c) STI-9, and (d) STI-12 in the study region (covered both Maizdi Court and Hatiya sub-districts) for the period 2002–2017. The *p-value* of the significance of the trend from the Kendall test is shown. The symbol ‘ns’ indicates the insignificance of the trend.

Like the assessment of temporal trends of SPEI values, using STI values, we also assessed the temporal trends of climatic conditions of 4 time scales (i.e., STI-3 to STI-12) for the period 2002–2017 in our study region (Fig 7). The STI values of all time scales showed decreasing trends, of which STI-3, STI-6, and STI-12 values significantly decreased over the past 16 years (all p < 0.05; Fig 7A, 7B and 7D). These decreasing trends in STI values suggest an increasing drier climatic conditions. The significance of these trends indicates that the studied regions experienced increasing intensity of drought.

**Fig 8:**
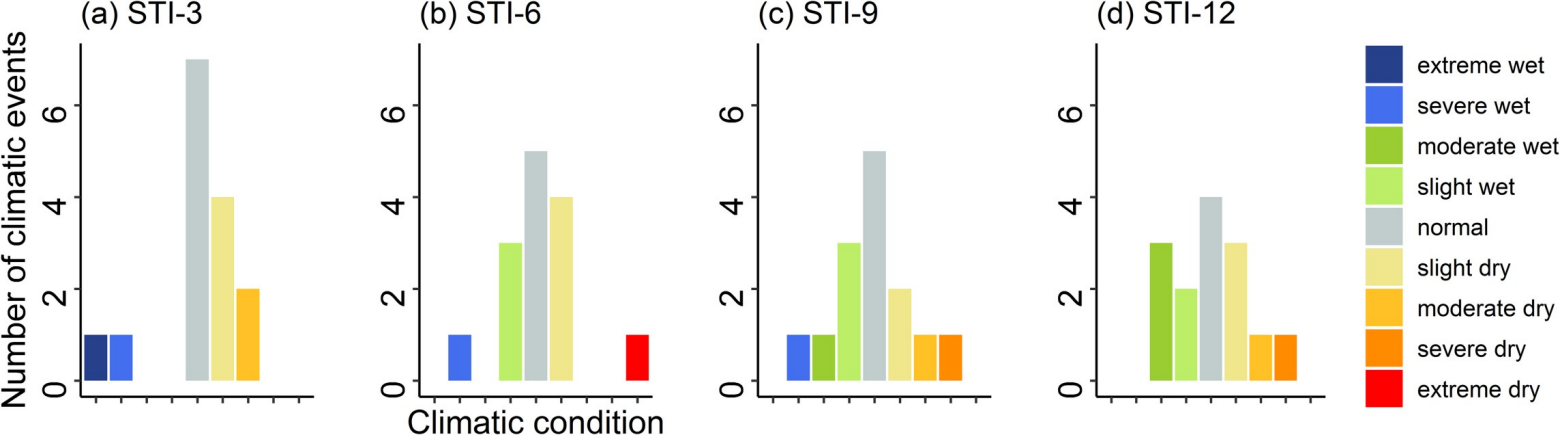
The frequency of 9 climatic conditions (extreme wet, severe wet, moderate wet, slight wet, normal, mild dry, moderate dry, severe dry, and extreme dry) based on Standardized Terrestrial Water Storage Index (STI) values for 4 time scales: (a) STI-3, (b) STI-6, (c) STI-9, and (d) STI-12 observed in the study area.

The assessment of the frequency of climatic conditions utilizing STI values in the study region unveiled a variety of occurrences, encompassing both wet and dry events, ranging from extreme wet to extreme dry conditions (Fig 8). The study region experienced 6 dry and 2 wet events for STI-3 values, and 5 dry and 4 wet events for STI-6 values. There were 4 dry and 5 wet events for STI-9 and 5 dry and 5 wet events for STI-12 values in the study area (Fig 8).

**Fig 9:**
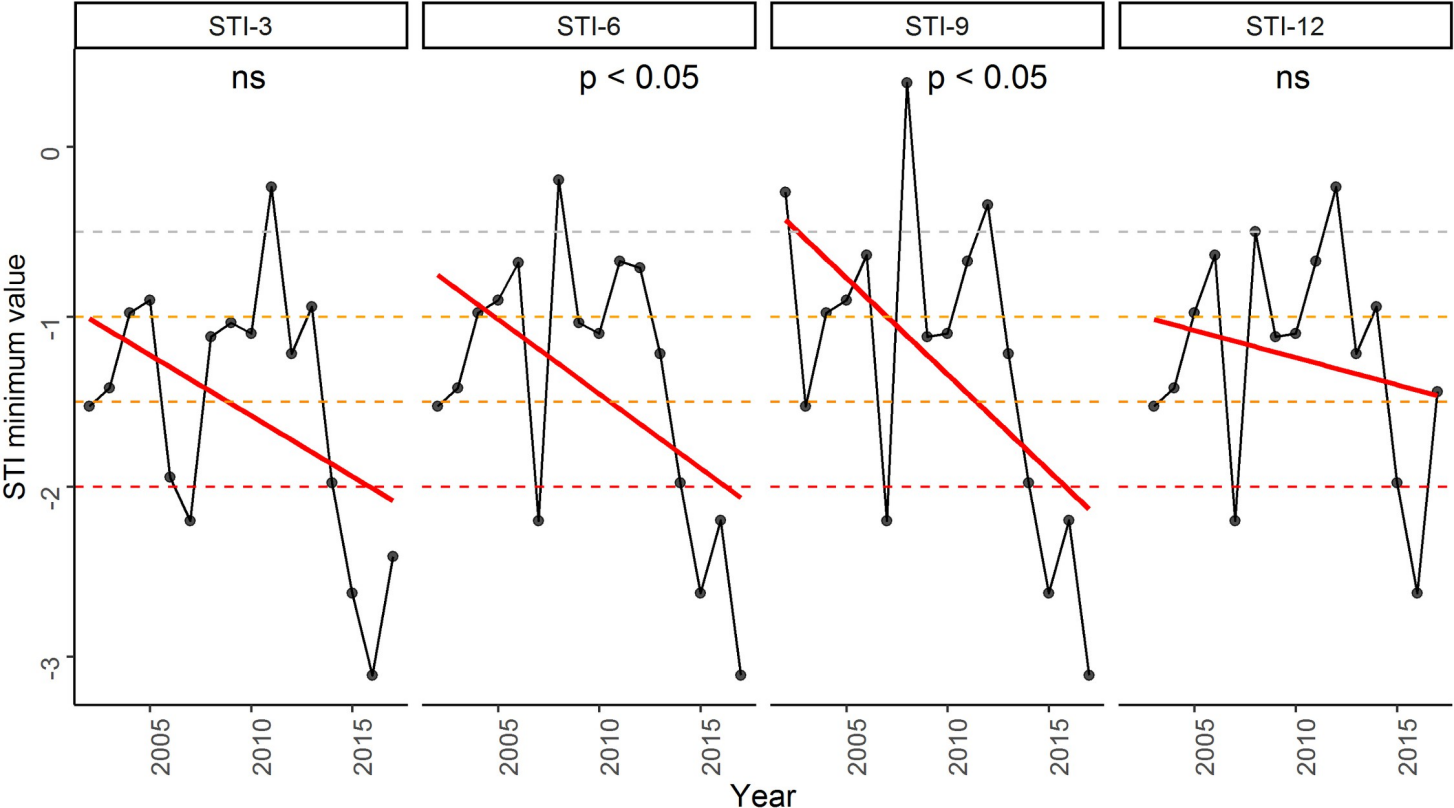
Temporal trends of Standardized Terrestrial Water Storage Index (STI) minimum values of 4 time scales (i.e., STI3, STI6, STI9, and STI12) in the study area. The *p-value* of the significance of the trend from the Kendall trend test is shown. The symbol ‘ns’ indicates the insignificance of the trend.

Similar to the temporal trend of minimum SPEI values (Fig 6), we further assessed the temporal trend of the intensity of dry climatic conditions using minimum STI values in our study area (Fig 9). This minimum STI values exhibited decreasing trends for all STI values, of which STI-6 and STI-9 minimum values significantly decreased for the period 2002–2017 (both *p* < 0.05, Fig 9). The decreasing trends of the remaining two STI minimum values (i.e., STI-3 and STI-12) were not significant (*p* > 0.05, Fig 9). The significant decreases in the minimum STI-6 and STI-9 values suggest a declining intensity of dry climatic conditions over the past 16 years. This indicates a potential decrease in the severity of dry spells or drought events.

Understanding drought frequency and intensity is critical for the sustainable management of agriculture. Bangladesh has experienced several historical droughts that have severely affected agricultural crop production. In our study, we assessed both meteorological droughts (using SPEI values) and hydrological droughts (using STI values) to explore the temporal trends and frequency of climatic conditions ranging from extreme wet to extreme dry conditions (Figs 3–9). The observed increasing intensity of growing season dry climatic conditions for both drought indices (Figs 3 and 7) in our study suggests that the coastal regions of Bangladesh are vulnerable to extreme droughts. These findings are consistent with several studies conducted in seven climatic zones [17], the western region [74], and the northwestern region [18]. For example, using SPEI values, Rahman et al. [17] reported that growing season droughts (SPEI-3 and SPEI-6) are more prevalent in Bangladesh compared to annual or biennial droughts (SPEI-12 and SPEI-24). While previous research has primarily focused on drought assessment in the western regions (northeast and northwest) [18, 31], our findings for southeastern sub-districts provide important information about the increasing occurrence of droughts in coastal regions. Therefore, it is crucial to introduce drought-adaptive mechanisms for the sustainable management of agriculture and other livelihoods in these coastal regions.

The increasing drought intensity in our studied regions is further exacerbated by the growing frequency of drought events (Figs 5 and 8). The analysis of growing season climatic conditions using SPEI-3 values revealed that there was 1 dry event every 4 years at Maizdi court station (over 1952–2019) and 1 dry event every 3.5 years at Hatiya station (over 1966–2020) (Fig 5). When hydrological drought is considered, it is observed that the frequency of growing season dry conditions was 2.5 in the study region for the period 2002–2017 (Fig 7). The recorded frequency of dry events in the recent 16 years highlights the increasing occurrence of drought events in our study regions. The increasing frequency and intensity of dry events based on SPEI and STI analysis supports the findings obtained from the thematic and content analysis of the qualitative data, where respondents in FGD and KII expressed their concern of rising growing season drought and drought-induced crop productivity loss.

The coastal region of Bangladesh is vulnerable to tropical cyclones, storm surges, floods, rising sea levels, salinity intrusion, and river bank erosion. These natural calamities have been responsible for widespread destruction of agricultural lands and crop productivity [12, 57]. In recent decades, droughts have been causing an emerging threat to the lives and livelihoods of these regions [31]. These emerging climate-induced stresses, including heatwaves and droughts, will have significant impacts on crop productivity, which will affect food security in these geographically challenged and disaster-prone coastal communities in Bangladesh. Therefore, introducing climate-adaptive techniques, including drought- and heat-resilient crops and crop diversification, is vital for the sustainable production of crops and for ensuring dietary diversity among the disadvantaged communities in the coastal regions. Despite the knowledge of several climate-adaptive agricultural techniques among the respondents of FGD, farmers in the coastal region are emphasizing the need for attention from the government and NGOs in terms of capacity-building training, awareness programs, availability of stress-resilient seeds, subsidies for organic fertilizers, and support for irrigation. These findings highlight the farmers’ recognition of the importance of implementing climate-resilient practices and their desire for external support to enhance their adaptive capacity. Therefore, government agencies and NGOs must prioritize these needs and provide the necessary resources and assistance to empower farmers in the coastal region to manage the challenges posed by climate change.

#### Coupling the qualitative and quantitative findings

Understanding the perceptions of farmers and agriculture-focused service providers and stakeholders regarding crop cultivation, climate change, and climate-adaptive crop cultivation practices is crucial for effective crop production in coastal regions. Using 2 Participatory Rural Appraisal (PRA) tools (i.e., FGD and KII), respondents’ observations and experiences regarding traditional agricultural practices, modifications in farming techniques under changing climatic conditions, and challenges in food security have been documented for the geographically under-improvised communities in 2 coastal sub-districts. The reported agricultural practices, emerging threats to crop production, adjustment in crop cultivation, and future challenges to agriculture and food security in this study highlight that farmers in the studied region have been struggling with climate-induced stresses, such as drought during crop growing season. Combining qualitative findings with quantitative analysis is of great importance to validate the respondents’ perceptions and experiences, which we did using climate data. The documented qualitative findings of the increasing occurrence of growing-season drought, as stated by FGD and KII respondents, are consistent with the accelerating intensity and frequency of growing-season drought observed in the analysis of meteorological drought (SPEI-3 and SPEI-6) and hydrological drought (STI-3 and STI-6). By combining qualitative and quantitative approaches, we were able to triangulate the data and enhance the robustness of our findings. This integrated approach strengthens the validity of our conclusions and underscores the urgency of implementing drought adaptation measures in the coastal regions.

## Conclusions

Climate change has been widely reported as having a detrimental effect on crop productivity, resulting in a decrease in agricultural yields. This impact is especially pronounced in coastal regions, which are highly susceptible to the adverse effects of tropical cyclones, storm surges, droughts, and flooding. In this context, understanding farmers’ and stakeholders’ perceptions regarding drought-induced crop loss, their adoption of adaptive techniques, and the challenges they face in ensuring food security becomes crucial for promoting climate-resilient crop cultivation practices and sustainable agricultural production.

Using two common participatory rural appraisal tools (i.e., FGD and KII), we assessed farmers’ and stakeholders’ perceptions regarding the challenges and adaptive practices in crop cultivation under climate change in two coastal sub-districts in Bangladesh. The intensified and frequent droughts in crop growing season and untimely precipitation were reported as the most pressing issues by the participants in FGD and KII. Despite these climate-induced stresses, farmers have adopted several adaptive practices in crop cultivation. Participants in FGDs reported that (i) cultivation of heat- and drought-resilient crop varieties (e.g., BINA 7 paddy, maize, sunflower, and soybean), (ii) storage of rainwater in ponds for irrigation purposes, (iii) use of mulching techniques to conserve moisture in the soil, (iv) application of organic fertilizers (e.g., vermicompost and cowdung) to improve soil health, and (v) adoption of short-duration crop cultivation to better adapt to changing climatic conditions have grown popularity as a measure of agricultural resilience under increasing frequency and intensity of climate change. The qualitative results also revealed that farmers in the studied sub-districts have been experiencing significant challenges in achieving dietary diversity and access to nutritional foods. These challenges arise from various factors, including poverty, lack of awareness, and limited crop diversification. The impact of climate-related challenges further exacerbates the situation, creating additional obstacles to food security and nutrition.

Given the respondents’ concern about drought-induced crop loss, we further assessed temporal trends of climatic conditions in respective sub-districts. Both SPEI and STI values exhibited an increasing dry climatic condition in the studied region, which supported and validated the qualitative findings. The convergence of qualitative and quantitative evidence highlights the necessity for proactive and targeted interventions to enhance agricultural resilience and ensure sustainable food production in a changing climate.

This study provides valuable insights into the challenges faced by farmers in coastal regions of Bangladesh. It is crucial to foster collaboration among policymakers, researchers, agricultural extension services, and farmers’ organizations to ensure successful policy implementation and knowledge sharing. Prioritizing capacity-building initiatives, training programs, and information dissemination campaigns can increase farmers’ awareness of climate change impacts and adaptation strategies. Policymakers and stakeholders should focus on implementing targeted measures to support farmers in adopting climate-resilient practices, improving access to diverse and nutritious food, and addressing the socio-economic barriers that hinder agricultural adaptation.

## Supporting information

S1 File SPEI, STI climate data and KII, FGD transcribes. (XLSX)

S2 File Questionnaire for participant. (PDF)
