# Supplementary material for: Climate-adaptive strategies for enhancing agricultural resilience in southeastern coastal Bangladesh: Insights from farmers and stakeholders
Source: PLoS One. 2024 Jun 21;19(6):e0305609. doi: 10.1371/journal.pone.0305609 (PMC11192385; doi:10.1371/journal.pone.0305609)
Supplement: S2 File — (PDF) [file pone.0305609.s002.pdf]

SI2

Climate-adaptive strategies for enhancing agricultural resilience in southeastern coastal Bangladesh: Insights from farmers and stakeholders

Md Abdullah Al Mamun[1,2], Jianfeng Li[1,3,*], Aihong Cui[1], Raihana Chowdhury [2], Md Lokman Hossain[1,4,*]

[1]Department of Geography, Hong Kong Baptist University, Hong Kong, China

[2]Department of Food Technology and Nutrition Science, Noakhali Science and Technology University, Sonapur-3814, Noakhali, Bangladesh

[3]Department of Geography and Resource Management, The Chinese University of Hong Kong, Hong Kong, China

[4]Department of Environment Protection Technology, German University Bangladesh, Gazipur, Bangladesh

[*] Corresponding author's email: J. Li (jianfengli@cuhk.edu.hk) and M.L. Hossain (lokmanbbd@gmail.com)

Questionnaire for participant

Date of Interview: **Sample** ☐

A. Personal Information:

1. Name: ....

2. Affiliated Organization: …………………………………………………………...

B. KII Questions:

1. What is the effect of climate change in this area?
2. What are the impacts of climate change on agriculture in this area?
3. Can you give me any ideas regarding Climate adaptive Agriculture?
4. Do you know which institutions are promoting Climate adaptive Agriculture? How?
5. What kinds of barriers do farmers face to adopt adaptive Agriculture?
6. What kind of barriers do farmers face to ensuring better crop production in this area?
7. Do you give your opinion about food availability and accessibility in this area?
8. Would you share the food and nutrition situation in this area?

C. FGD Questions

1. What kind of climate change are you all currently experiencing, and how does it affect farming?
2. Can we share our concept and practice regarding Climate adaptable agriculture in this area?
3. What is the current situation of food and nutrition security in this area, and what barriers do farmers face to ensuring food security in this area?
